# The *SlZRT1* Gene Encodes a Plasma Membrane-Located ZIP (Zrt-, Irt-Like Protein) Transporter in the Ectomycorrhizal Fungus *Suillus luteus*

**DOI:** 10.3389/fmicb.2017.02320

**Published:** 2017-11-28

**Authors:** Laura Coninx, Anneleen Thoonen, Eli Slenders, Emmanuelle Morin, Natascha Arnauts, Michiel Op De Beeck, Annegret Kohler, Joske Ruytinx, Jan V. Colpaert

**Affiliations:** ^1^Environmental Biology, Centre for Environmental Sciences, Hasselt University, Hasselt, Belgium; ^2^Biomedical Research Institute, Hasselt University, Hasselt, Belgium; ^3^Institut National de la Recherche Agronomique, Laboratoire d’Excellence ARBRE, UMR 1136, Université de Lorraine Interactions Arbres/Microorganismes, Champenoux, France

**Keywords:** *Suillus luteus*, mycorrhiza, zinc transporter, zinc homeostasis, zinc deficiency, metal uptake

## Abstract

Zinc (Zn) is an essential micronutrient but may become toxic when present in excess. In Zn-contaminated environments, trees can be protected from Zn toxicity by their root-associated micro-organisms, in particular ectomycorrhizal fungi. The mechanisms of cellular Zn homeostasis in ectomycorrhizal fungi and their contribution to the host tree’s Zn status are however not yet fully understood. The aim of this study was to identify and characterize transporters involved in Zn uptake in the ectomycorrhizal fungus *Suillus luteus*, a cosmopolitan pine mycobiont. Zn uptake in fungi is known to be predominantly governed by members of the ZIP (Zrt/IrtT-like protein) family of Zn transporters. Four ZIP transporter encoding genes were identified in the *S. luteus* genome. By in silico and phylogenetic analysis, one of these proteins, SlZRT1, was predicted to be a plasma membrane located Zn importer. Heterologous expression in yeast confirmed the predicted function and localization of the protein. A gene expression analysis via RT-qPCR was performed in *S. luteus* to establish whether *SlZRT1* expression is affected by external Zn concentrations. *SlZRT1* transcripts accumulated almost immediately, though transiently upon growth in the absence of Zn. Exposure to elevated concentrations of Zn resulted in a significant reduction of *SlZRT1* transcripts within the first hour after initiation of the exposure. Altogether, the data support a role as cellular Zn importer for SlZRT1 and indicate a key role in cellular Zn uptake of *S. luteus*. Further research is needed to understand the eventual contribution of SlZRT1 to the Zn status of the host plant.

## Introduction

Zinc (Zn) is an essential micronutrient required by all living organisms (Eide, 2006). Due to its unique set of chemical properties, Zn plays both a functional and a structural role in many proteins. The metal is characterized by a small radius and has thus a highly concentrated charge (Clemens, 2006a). Furthermore, Zn is a Lewis acid with a high affinity for ligands with sulfur- (S), nitrogen- (N), and oxygen- (O) containing functional groups (Clemens, 2006a). Due to its full d-subshell, Zn is also able to interact with these ligands more flexibly than other transition metals (Berg and Shi, 1996). By supporting a tetrahedral coordination geometry, Zn allows proteins to quickly shift conformations in biological reactions. These properties combined with its lack of redox activity, explain why Zn is a valuable structural element (e.g., Zn finger proteins) and a catalyst for many enzymes (e.g., hydrolytic enzymes) (Frausto da Silva and Williams, 2001). However, the same properties that make Zn an indispensable nutrient can also induce toxicity (Clemens, 2006b). When present in excess, Zn can cause protein damage and inactivation by uncontrolled high-affinity binding to functional groups within proteins (Clemens, 2006b). For this reason, it is necessary for living cells to tightly regulate Zn concentrations in the cytoplasm. The physiological concentration range of Zn between deficiency and toxicity is extremely narrow and organisms are consequently equipped with a number of homeostatic mechanisms to tightly regulate cytoplasmic Zn concentrations (Eide, 2006). Especially transporter proteins play a crucial role in maintaining Zn homeostasis (Eide, 2006).

In eukaryotes most of the Zn transport is achieved by two protein families: the ZIP (Zrt/Irt-like protein) and CDF (Cation Diffusion Facilitator) transporter families (Gaither and Eide, 2001). Proteins belonging to the ZIP transporter family increase cytoplasmic Zn levels by transporting Zn across the plasma membrane or by mobilizing stored Zn from intracellular compartments. Whereas members of the CDF family transport Zn in the direction opposite to that of the ZIP proteins. Efflux or compartmentalization of Zn is promoted by transporting Zn from the cytoplasm into the lumen of an organelle or out of the cell (Gaither and Eide, 2001).

Transporters belonging to the ZIP family typically possess 5 to 8 transmembrane domains (TMDs). The protein sequence is most conserved in TMD IV and the region adjacent to TMD IV (Eng et al., 1998). The ZIP family can be divided into four subfamilies based on a higher degree of sequence similarity: the ZIP I, ZIP II, GufA and LIV-I subfamily (Guerinot, 2000; Gaither and Eide, 2001). Members of the ZIP family are well-studied in *Saccharomyces cerevisiae*, which is an excellent fungal model system to investigate Zn uptake and efflux (Zhao and Eide, 1996). Currently, four ZIP Zn transporter genes have been identified in baker’s yeast: *ZRT1, ZRT2, ZRT3*, and *YKE4*. The yeast *ZRT1* gene was the first influx Zn transporter gene from any organism to be characterized at the molecular level (Zhao and Eide, 1996). The *ZRT1* gene encodes a high-affinity Zn uptake system induced by Zn limitation, whereas the ZRT2 transporter corresponds to a low-affinity uptake system that is active in Zn repleted cells (Eide, 1996; Zhao and Eide, 1996). Zn uptake in yeast is predominantly governed by these two plasma membrane-located transporters (Eide, 1996; Zhao and Eide, 1996). Both transporters are included in the ZIP I subfamily (Gaither and Eide, 2001). The third characterized yeast ZIP protein, ZRT3, belongs to the GufA ZIP subfamily (Gaither and Eide, 2001). This transporter localizes to the vacuolar membrane and mobilizes Zn under Zn deficiency (MacDiarmid et al., 2000). Lastly, Kumánovics et al. (2006) characterized YKE4, a bidirectional Zn transporter in the endoplasmic reticulum (ER) of *S. cerevisiae*, which regulates Zn concentrations in the ER and cytoplasm. YKE4 is a LIV-I subfamily transporter (Gaither and Eide, 2001).

Additional to the research in yeast, other ZIP transporters and mechanisms of Zn homeostasis in fungi are primarily characterized and studied in human fungal pathogens. Membrane Zn importers of the ZIP I subfamily have been shown to be crucial for the acquisition of Zn and the virulence of several human pathogenic fungi (Crawford and Wilson, 2015). This was observed in *Candida albicans* for the Zn transporter CaZRT1 (Citiulo et al., 2012), in *Cryptococcus neoformans* for CnZIP1 (Do et al., 2016) and in *Aspergillus fumigatus* for AfZrfC (Amich et al., 2014). These transporters enable pathogenic fungi to overcome Zn deficiency within the Zn-limited host environment (Jung, 2015). Zn and fungal ZIP transporters are therefore considered to be key players in this kind of pathogenic host-microbe interactions.

In the current study we aim to identify plasma membrane localized Zn importers and their role in cellular Zn homeostasis in the ectomycorrhizal fungus *Suillus luteus*. Ectomycorrhizae are mutualistic host-microbe interactions between tree roots and ectomycorrhizal fungi. The mycobiont offers the tree a balanced nutrient supply in exchange for photosynthetic sugar (Martin et al., 2016). Although Zn is not expected to be a key regulator of ectomycorrhizal development nor to be extremely scarce at the symbiotic interface, availability of this element may have an impact on the fitness of both individual symbiotic partners and the mutualism in particular environments. Micronutrient deficiencies are rarely observed in natural forests but severe Zn deficiency in tree plantations has been reported previously (Thorn and Robertson, 1987; Boardman and McGuire, 1990). Moreover, trees are sensitive to high soil Zn concentrations. We previously demonstrated that well-adapted ectomycorrhizal fungi can protect host trees from Zn toxicity when Zn is present in excess (Adriaensen et al., 2004, 2006). An improved knowledge on the mechanisms of cellular Zn homeostasis in ectomycorrhizal fungi, going beyond the general focus on detoxification by vacuolar sequestration and including Zn uptake and deficiency, will be the first step toward a better understanding of the contribution of ectomycorrhizal fungi to host tree Zn homeostasis.

## Materials and Methods

### Fungal Strains and Culture Conditions

The monokaryotic *S. luteus* isolate UH-Slu-Lm8-n1 (Kohler et al., 2015) and the dikaryotic isolate UH-Slu-P4 (Colpaert et al., 2004) were used in this study. Cultures were maintained on solid Fries medium according to Colpaert et al. (2004). Preceding Zn exposure assays, 1-week-old exponentially growing mycelia were harvested and liquid cultures were initiated and maintained according to Nguyen et al. (2017). Three gram of spherical mycelia grown for 1 week in liquid culture were transferred to petri dishes containing 25 ml modified liquid Fries medium supplemented with 0, 20, 500, or 1000 μM ZnSO_4_⋅7H_2_O. These Zn concentrations were chosen to induce Zn deficiency, Zn sufficiency and mild Zn toxicity (Ruytinx et al., 2017). The petri dishes were incubated on a shaking incubator at 23°C. Metal exposure was performed in triplicate. Mycelia (400 mg) were sampled at 0, 1, 2, 4, 8, and 24 h after initiation of exposure, flash frozen in liquid nitrogen and stored at -70°C.

### ZIP Identification and Phylogenetic Tree Construction

The *S. luteus* reference genome was searched for ZIP transporter encoding genes. A BLASTp search using characterized fungal ZIP transporters (Supplementary Table 1) and a Pfam domain search were performed at the *S. luteus* genome portal at MycoCosm of the Joint Genome Institute (JGI)^1^ (Grigoriev et al., 2012; Kohler et al., 2015). Full-length amino acid sequences of previously characterized ZIP transporters were obtained from the transporter classification database^2^, the Swissprot database^3^ and the National Center for Biotechnology Information (NCBI) server^4^. All sequences, including the newly identified *S. luteus* ZIP sequences, were aligned with the Multiple Alignment using Fast Fourier Transform (MAFFT) alignment logarithm version 7 (Katoh and Standley, 2013) and imported into the Molecular Evolutionary Genetics Analysis (MEGA) package version 6.06 (Tamura et al., 2013). A phylogenetic tree was constructed using the neighbor-joining (NJ) method (Poisson correction model for distance computation) to infer evolution of the identified *S. luteus* ZIP transporters and to predict their function more precisely.

### Cloning of *SlZRT1*

Total RNA was extracted from *S. luteus* mycelium ground in liquid nitrogen using the RNeasy Plant Mini kit (Qiagen, Germany) and a cDNA library was constructed using the SMARTer cDNA synthesis kit (Clontech, United States) according to the manufacturer’s instructions. Specific primers were designed for amplification of the full-length coding sequence of *SlZRT1* (left: 5′ CCTCAAACTATGTCAGATTTAAATT 3′; right: 5′ TGCCCAACGCCCCAGGAGC 3′). The PCR reaction contained: 10x High Fidelity PCR buffer, 0.2 mM dNTP-mixture, 2 mM MgSO_4_, 0.2 μM *SlZRT1* forward and reverse primer, 5 ng cDNA and 0.5 U Platinum Taq High Fidelity DNA polymerase (Invitrogen, United States). RNase-free water was added to obtain a final reaction volume of 30 μl. The following PCR cycling conditions were used: 2 min at 95°C; 35 cycles of 30 s at 95°C + 30 s at 56°C + 1 min at 68°C, and 1 cycle of 3 min at 68°C. 5 μl of the PCR product was visualized on an agarose gel to verify the reaction specificity and the length of the amplicon. The remaining 25 μl PCR product was purified using the GeneJET PCR Purification Kit (ThermoScientific, United States). The purified PCR-product was cloned into the gateway entry vector pCR8/GW/TOPO (Invitrogen) and subsequently transferred by the Gateway LR-clonase II Enzyme Mix (Invitrogen) to destination vectors pYES-DEST52 (Invitrogen, United States) and pAG426GAL-ccdB-EGFP (Alberti et al., 2007) for functional analysis in yeast. The insert was sequenced in both directions to verify correct orientation and fusion.

### Yeast Mutant Complementation and Subcellular Localization

*SlZRT1* was heterologous expressed in *S. cerevisiae*. Yeast strains used are CM30 (MATα, ade6, can1-100, his3-11, 15 leu2-3, trp1-1, ura3-52) and CM34 or Δzrt1Δzrt2 (CM30, zrt1::LEU2, zrt2::HIS3) (MacDiarmid et al., 2000). Yeast cells were transformed according to the LiAc/PEG method as described by Gietz and Woods (2002). Transformed yeast cells were selected on synthetic defined medium without uracil [SD-URA; 0.7% w/v yeast nitrogen base (Difco), 2% w/v D-glucose, and 0.2% w/v Yeast Synthetic Drop-out Mix without uracil (Sigma)]. Plates were incubated at 30°C.

For metal tolerance assays, transformed yeasts were grown to mid log phase (OD_600_ ± 1.5) in liquid SD-URA medium with 2% w/v D-galactose instead of D-glucose (induction medium). Yeast cells were pelleted, washed with sterile distilled water, and adjusted to OD_600_ = 1. A 1/10 dilution series was prepared (10^0^, 10^-1^, 10^-2^, and 10^-3^). Drop assays were performed for three independent yeast clones on SD-URA control induction medium (1 mM Zn) and induction medium supplemented with 50, 100, or 200 μM ethylenediaminetetraacetic acid (EDTA) (MacDiarmid et al., 2000). For subcellular localization of SlZRT1::EGFP fusion proteins, yeast transformants were grown to mid-log phase OD_600_ = 1 on induction medium. Plasma membrane of the cells was stained at 0°C by FM4-64 (Molecular Probes, Invitrogen) according to Vida and Emr (1995). Afterward, a 3 μl droplet of yeast cells was analyzed at 0°C with a Zeiss LSM 510 META laser scanning confocal microscope (Carl Zeiss, Jena, Germany), using a Zeiss 40x NA1.1 water immersion objective (C-Apochromat 40x/1.1 W Corr., Carl Zeiss). Enhanced green fluorescent protein (EGFP) fluorescence analysis was performed with the 488 nm excitation line of an argon-ion laser and a band-pass 500–550 nm emission filter. FM4-64 (ThermoFisher) fluorescence analyses were performed with a 543 nm HeNe laser and a long-pass 560 nm emission filter. Image processing was carried out with ImageJ (NIH, Bethesda, MD, United States) software.

### Zn Content Analysis of Transformed Yeast

Transformed yeast cells were cultured at 30°C in liquid induction medium without Zn until culture saturation. Three rounds of Zn deprivation were completed by re-inoculating 0.5 ml of saturated yeast suspension to new Zn-less induction medium. Zn-starved cells were grown to mid log phase (OD_600_ ± 1.5) and diluted to OD_600_ = 1. One ml of yeast suspension was added to Erlenmeyer flasks containing 20 ml liquid induction medium without Zn and medium supplemented with 500 μM Zn (repletion). Zn treatments were performed for five independent yeast clones. Cultures were allowed to grow for 24 h at 30°C. Yeast cells were collected by centrifugation, washed three times with 20 mM PbNO_3_ and milli-Q water. Afterward cells were resuspended in 0.5 ml of milli-Q water, frozen (-20°C) and lyophilized. Lyophilized cells were acid digested (HNO_3_/HCl) and Zn content was determined by inductively coupled plasma optical emission spectrometry (ICP-OES).

### RNA Extraction, cDNA Synthesis, and qPCR

Total RNA was extracted from mycelium pulverized in liquid nitrogen using the RNeasy Plant Mini Kit (Qiagen). The TURBO DNA-free kit [Invirtogen (Life Technologies), United States] was used to perform a desoxyribonuclease (DNase) treatment to remove residual genomic DNA. RNA integrity and absence of DNA contamination was verified via agarose gel analysis. RNA concentration and purity were evaluated on a NanoDrop ND-1000 Spectrophotometer (Isogen Life Science, the Netherlands). One μg of each RNA sample was converted to cDNA with the Primescript RT Reagent Kit (Perfect Real Time) (TaKaRa Clontech, United States). A 10-fold dilution of the cDNA was prepared in 1/10 diluted Tris-EDTA (TE) buffer and stored at -20°C.

Real-time reverse transcription polymerase chain reaction (qRT-PCR) was performed in a 96-well optical plate with an ABI PRISM 7500 Fast Real-Time PCR System (Life Technologies) according to Ruytinx et al. (2016). A *SlZRT1* specific primer pair was designed using Primer3 (Rozen and Skaletsky, 2000) (5′ GCCAAACGGACAAACTGG 3′; 5′ GACAGGCACGGAGATGAAAG 3′; efficiency = 92.1%). Data were expressed relative to the sample with the highest expression level via the formula 2^-(Ct-Ctmin)^ and normalized using five reference genes (*TUB1, ACT1, GR975621, AM085296*, and *AM085296*). Reference genes were selected previously by Ruytinx et al. (2016) and their stability was confirmed within the current experimental conditions using GeNorm (Vandesompele et al., 2002). A normalization factor was calculated as the geometric mean of the relative expression levels of the reference genes. Mean values of four biological replicates were calculated, rescaled to the control condition (20 μM Zn) within each time point and log2 transformed. A two-way analysis of variance (ANOVA) followed by a Tukey’s HSD test was run in “R” version 3.2.2 (R Core Team, 2015) to assess differences in *SlZRT1* expression level.

## Results

### Identification of a ZIP Transporter in *S. luteus*

**FIGURE 1:**
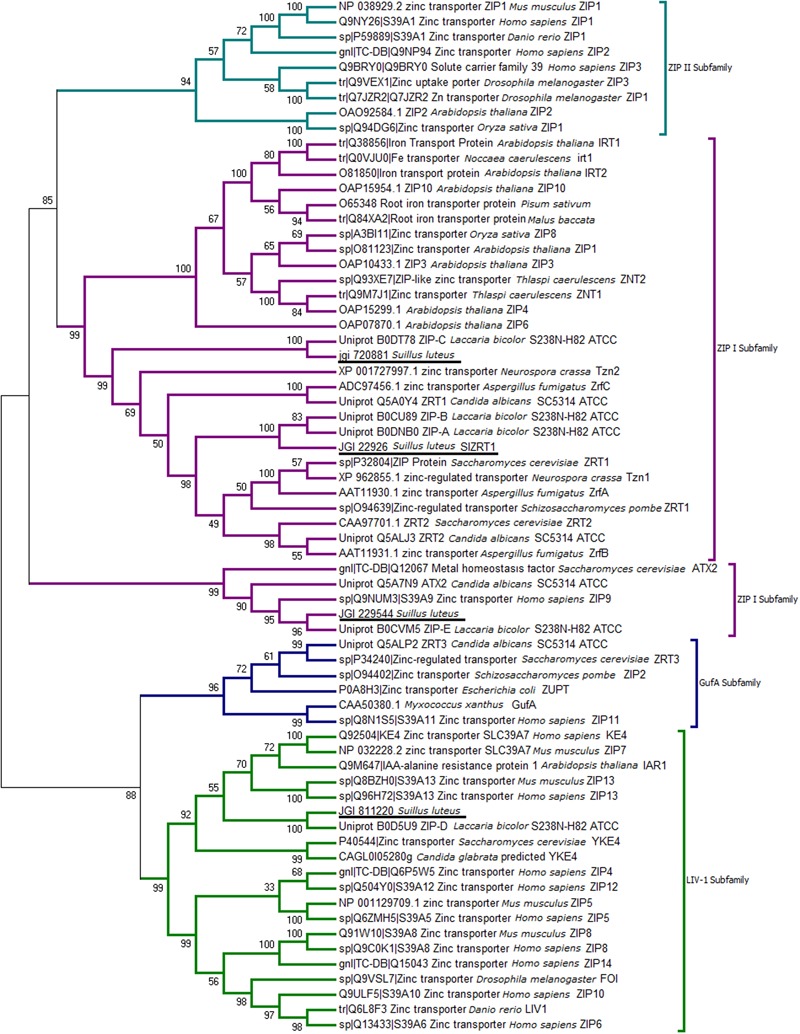
Neighbor-joining (NJ) tree of the Zrt- Irt- like protein (ZIP) family with sequences from selected fungi and characterized ZIP proteins from other species. Sequences were aligned by the Multiple Alignment using Fast Fourier Transform (MAFFT version 7) algorithm, bootstrap values (1000 replicates) are indicated, and *Suillus luteus* sequences are underlined.

The BLASTp search of the *S. luteus* genome (UH-Slu-Lm8-n1 v2.0) with characterized fungal ZIP transporters identified four *S. luteus* genes (protein IDs 720881, 22926, 229544, and 811220) predicted to encode ZIP proteins. A NJ phylogenetic tree including previously characterized ZIP transporters was constructed to predict the function of the newly identified *S. luteus* genes. The different ZIP subfamilies are well-supported in the tree as indicated by high bootstrap values (>90; **Figure 1**). Three of the identified *S. luteus* proteins (IDs 720881, 22926, and 229544) cluster within the ZIP I subfamily. Within this subfamily, proteins with ID 720881 and 22926 cluster together with the *S. cerevisiae* Zn importers ScZRT1 and ScZRT2; the protein with ID 229544 clusters with *S. cerevisiae* ATX2, a Golgi transporter involved in manganese (Mn) homeostasis. The fourth identified *S. luteus* gene encodes a protein (ID 811220) clustering close to the *S. cerevisiae* YKE4 (ER localized Zn transporter) within the LIV-1 subfamily of ZIP transporters. We were not able to detect a member of the Guf A and ZIP II subfamily of ZIP transporters within the *S. luteus* reference genome.

**FIGURE 2:**
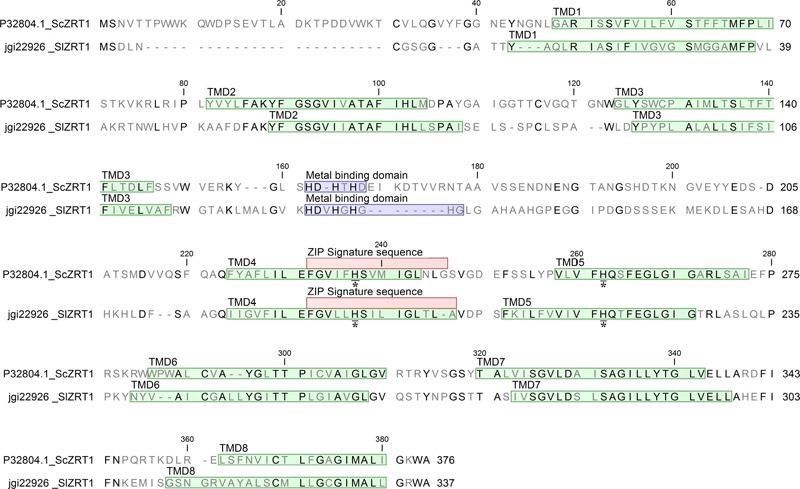
Alignment of SlZRT1 and ScZRT1 protein sequences. The eight transmembrane domains (TMDs) predicted by topology prediction program TMHMM2.0 are indicated in green, the putative metal binding domains in blue, the ZIP signature sequence as described by Eng et al. (1998) in red, and two conserved histidines with an asterisk (located in TMD4 and TMD5).

One identified *S. luteus* gene, encoding the protein with ID 22926 was selected for further analysis because of its high sequence similarity with the yeast Zn importers ScZRT1 and SpZRT1. Reciprocal BLASTp suggest the *S. luteus* protein to be orthologous to the high-affinity Zn importers ScZRT1 and SpZRT1 and was therefore named SlZRT1. *SlZRT1* is predicted to have a 1398 bp open reading frame with eight exons encoding a 338 amino acid polypeptide. The encoded peptide shows several characteristics that are typical for proteins belonging to the ZIP family (**Figure 2**). Eight TMDs were predicted by the topology program TMHMM and a long variable cytoplasmic loop is present between TMD3 and TMD4. A histidine rich motif HXX(HX)_3,_ suggested to function as Zn binding site, is present in the variable cytoplasmic loop of SlZRT1 and two other histidines that are typically conserved in ZIP transporters were identified (**Figure 2**). One of these conserved histidines is located in the conserved TMD4, which contains the ZIP signature sequence described by Eng et al. (1998). SlZRT1 matches 13 of the 15 amino acids of this ZIP signature sequence.

### Functional Analysis of *SlZRT1* in Yeast

**FIGURE 3:**
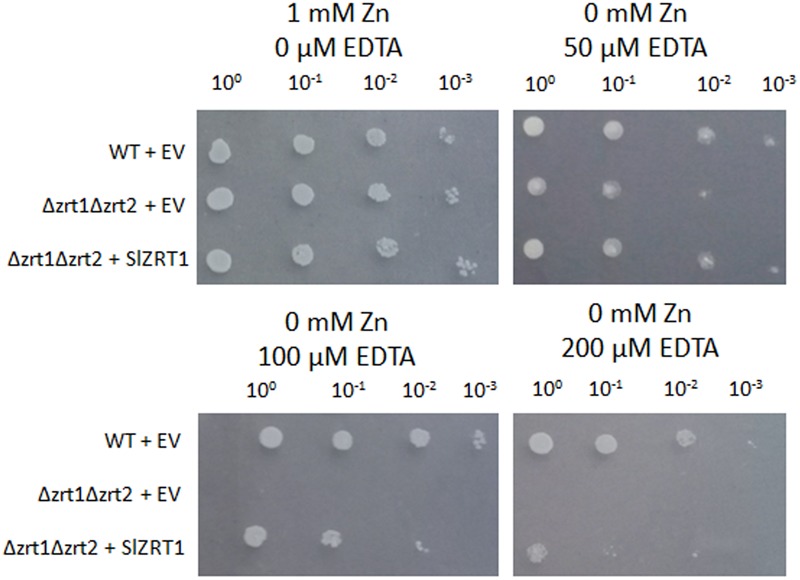
Functional complementation of the zinc-uptake-deficient yeast strain Δzrt1Δzrt2 by SlZRT1. Wild type (WT) and mutant yeast cultures with an OD_600_ = 1 were 10-fold serial diluted (10^0^, 10^-1^, 10^-2^, and 10^-3^) and spotted on control (1 mM Zn) or ethylenediaminetetraacetic acid (EDTA) supplemented synthetic drop-out (SD) medium. The WT strain was transformed with the empty vector (EV, pYES-DEST52; Invitrogen), the mutant strain Δzrt1Δzrt2 with either the EV or the vector containing *SlZRT1*. The experiment was carried out for three independent clones and pictures were taken after 4 days of growth.

**FIGURE 4:**
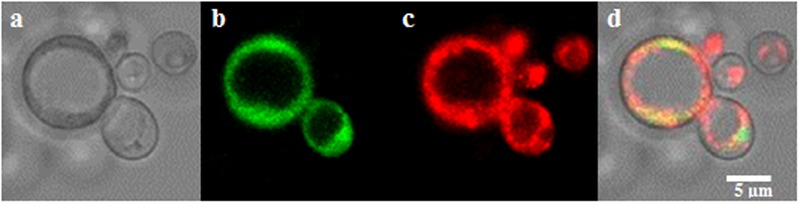
Localization of the SlZRT1:EGFP fusion protein to the plasma membrane in yeast **(a–d)**. **(a)** Bright field image, **(b)** EGFP fusion protein, **(c)** FM4-64 plasma membrane staining, and **(d)** merged images. SlZRT1:EGFP and FM4-64 plasma membrane staining co-localize.

*SlZRT1* was heterologous expressed in yeast to confirm that it encodes a plasma membrane-located ZIP Zn importer, which was predicted by the phylogenetic analysis. **Figure 3** and **Supplementary Figure 1** illustrate that transformation with *SlZRT1* partly restored the growth of the zinc-uptake-deficient yeast strain Δzrt1Δzrt2 on medium supplemented with different concentrations of EDTA. Transformation with the empty vector did not result in complementation of the Zn deficient phenotype (**Figure 3** and **Supplementary Figure 1**). Expression of the SlZRT1::EGFP fusion protein indicates a localization of SlZRT1 on the plasma membrane. Yeast cells transformed with SlZRT1::EGFP showed a bright green fluorescent ring surrounding the cells, which co-localized with FM4-64 plasma membrane staining (**Figure 4**).

### Zn and Fe Content Analysis of Transformed Yeast

**FIGURE 5:**
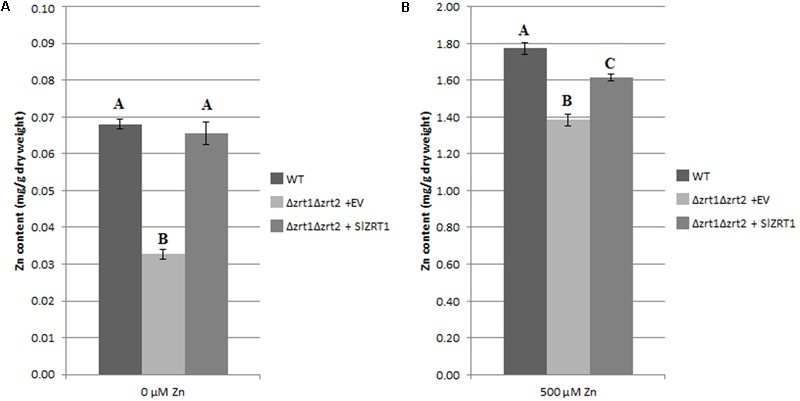
Intracellular Zn concentrations in transformed yeast cells **(A,B)**. The WT strain was transformed with the EV (pYES-DEST52, Invitrogen), the mutant strain with either the EV or the vector containing *SlZRT1*. Data are the average ± SE of five biological replicates, significant differences (*p* < 0.05) are indicated by different letters. **(A)** In control conditions (0 μm Zn), **(B)** after exposure to Zn (500 μM).

Zn and Fe content were measured in Zn starved (**Figure 5A**) and Zn replete (**Figure 5B**) yeast cells in order to obtain more insight into the function of SlZRT1. **Figure 5A** illustrates that Δzrt1Δzrt2 yeast mutants transformed with *SlZRT1* contained the same amount of Zn as the wild type (WT) yeast after starvation (0 μM Zn) while Δzrt1Δzrt2 mutants transformed with the empty vector had a significantly lower Zn content. Similarly, 24 h after Zn repletion (500 μM Zn) *SlZRT1* transformed yeast mutants and WT yeast accumulated significantly more Zn than empty vector transformed yeast mutants (**Figure 5B**). A small difference in Zn accumulation was observed between WT yeast cells and *SlZRT1* transformed Δzrt1Δzrt2 yeast cells. Additionally the Fe content in the yeast transformants was analyzed, since some ZIP transporters can also use Fe as a substrate. Yet, no significant differences in Fe content were observed among the yeast transformants exposed to 0 or 500 μM Zn (**Supplementary Figures S2A,B**).

### *SlZRT1* Gene Expression Analysis in *S. luteus*

**FIGURE 6:**
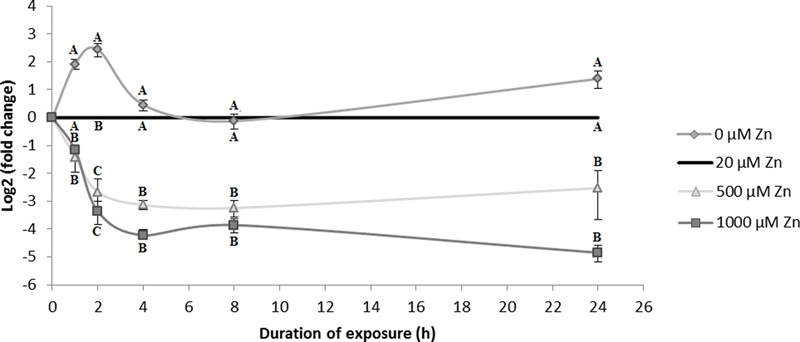
*SlZRT1* gene expression in *S. luteus* mycelium after 0, 1, 2, 4, 8, and 24 h exposure to different concentrations of Zn. Data are the average ± SE of three biological replicates and expressed as fold change to the control condition (20 μM Zn) within each time point. Mean values were log2 transformed. Significant differences (*p* < 0.05) within each time point are indicated by different letters.

In *S. luteus, SlZRT1* gene expression was determined at early time points (0, 1, 2, 4, 8, and 24 h) after exposure to different concentrations of Zn [0, 20 (control), 500, and 1000 μM] to assess the role of the *SlZRT1* in Zn homeostasis. Results clearly illustrate that mRNA levels of *SlZRT1* are dependent of external Zn concentration (**Figure 6**). Exposure to mildly toxic Zn concentrations (500 and 1000 μM) results in an almost immediate significant downregulation of *SlZRT1* gene expression. The expression patterns upon exposure to 500 and 1000 μM Zn are similar regardless differences in external Zn concentrations. In contrast, in the absence of external Zn, *SlZRT1* expression is quickly induced to reach a maximum level after 2 h, declines to control levels after 4 h and tends to be higher again in the long term (24 h).

## Discussion

Transition metals, such as Zn, Fe, Mg, are essential to all living organisms. However, when present in excess these metals may become toxic. To overcome metal toxicity, it is crucial for cells to tightly control cytoplasmic metal concentrations (Eide et al., 2005). Metal transporter proteins play a crucial role in the regulation of cytoplasmic metal concentrations and cellular metal homeostasis (Migeon et al., 2010). Among fungi, mechanisms involved in Zn homeostasis are mostly studied in *S. cerevisiae.* Transporters of the ZIP family were shown to be vital to prevent Zn deficiency in this species (Eide, 2006) and several other fungi (Kiranmayi et al., 2009; Jung, 2015). *S. cerevisiae* possess two plasma membrane localized Zn importers of the ZIP family (ScZRT1 and ScZRT2) and one tonoplast localized ZIP transporter (ScZRT3) for re-mobilization of vacuolar stored Zn. In the current study, we identified four ZIP transporter encoding genes in the genome of the ectomycorrhizal fungus *S. luteus*. Three of the newly identified proteins are members of the ZIP I subfamily of ZIP transporters, one belongs to the LIV-1 subfamily (**Figure 1**). With the exception of ScZRT3, a tonoplast transporter involved in Zn mobilization from the vacuole, homologs for all characterized *S. cerevisiae* ZIP transporters were identified within the *S. luteus* genome. So far, no homologs for the ScZRT3 protein have been identified in members of the Basidiomycota. Nevertheless, several basidiomycetes including *S. luteus* store excess Zn into their vacuoles (Sacky et al., 2016; Ruytinx et al., 2017). Transporters belonging to other protein families likely evolved in these species to re-mobilize stored Zn in absence of external environmental Zn. In accordance with what has been found in other fungi, there was no member of the ZIP II subfamily of ZIP transporters detected in *S. luteus*. This subfamily consists mainly of metazoan representatives (Guerinot, 2000).

Reciprocal BLASTp suggested the *S. luteus* protein with ID 22926, named SlZRT1 to be orthologous to the *S. cerevisiae* ScZRT1 transceptor. ScZRT1 functions as a high-affinity Zn uptake transporter and receptor (Schothorst et al., 2017). Together with its homolog, the plasma membrane transporter ScZRT2, ScZRT1 is responsible for Zn uptake in Zn deficient yeast cells (Gaither and Eide, 2001). SlZRT1 and ScZRT1 show 39% sequence identity. An important difference in the sequence of SlZRT1 and ScZRT1 is found within the putative Zn binding domain (histidine rich domain, HRD) localized within the cytoplasmic loop between TMD3 and TMD4 (**Figure 2**). SlZRT1’s binding domain (HDVHGHGHG) shows a HXX additional to the classical (HX)_3_ domain of ScZRT1 (HDHTHD). This difference might correspond to an altered affinity toward Zn and/or a modified function of the protein. Mutation of the histidines in the HRD of ScZRT1 results in a 70% reduction in the maximum uptake rate of ScZRT1 (V_max_), whereas the substrate concentration at which the reaction rate is half of V_max_ (K_m_) remains unaffected (Gitan et al., 2003). Also for other ZIP1 subfamily transporters a reduction in Zn uptake due to mutation of histidines in the HRD was observed (Mao et al., 2007) and some of these histidines are even necessary for the protein to be functional, i.e., able to transport Zn across the plasma membrane (Milon et al., 2006).

Heterologous expression and subcellular localization in yeast are common experimental procedures to study eukaryotic gene function and protein localization (Zhao and Eide, 1996; Mokdad-Gargouri et al., 2012). Heterologous expression of *SlZRT1* in the Δzrt1Δzrt2 yeast double mutant, which is defective in Zn uptake, resulted in an almost complete restoration of the phenotype (**Figure 3**) and SlZRT1::EGFP fusion proteins localize at the plasma membrane of yeast cells (**Figure 4**). These results support a role as plasma membrane localized Zn transporter for the SlZRT1 protein. However, kinetics of the transporter might be different from the ScZRT1 protein. SlZRT1 did not fully complement ScZRT1 as observed in the drop assays (**Figure 3**) and Zn starved *SlZRT1* transformed Δzrt1Δzrt2 yeast cells accumulate less Zn within 24 h after Zn replenishment than WT yeast cells do (**Figure 5**). No significant differences in Fe content were observed (**Supplementary Figure 2**), indicating a high Zn specificity of the transporter.

In yeast, *ScZRT1* expression is regulated both at the transcriptional and the post-transcriptional level by Zn (Gitan and Eide, 2000). Post-translationally, Zn induces the removal of ScZRT1 from the plasma membrane via ubiquitination (Gitan et al., 2003). After endocytosis the protein is degraded in the vacuole. This regulatory system ensures a rapid shutdown of Zn uptake in yeast cells exposed to high concentrations of Zn (Gitan and Eide, 2000). In *S. luteus SlZRT1* expression is regulated by excess Zn. *SlZRT1* expression level is significantly lower after exposure to potentially toxic concentrations of Zn (500 and 1000 μM) as compared to the control (20 μM) and this already 1 h after initiation of the exposure (**Figure 6**). In contrast, absence of external Zn results in a rapid accumulation of *SlZRT1* mRNA. Two hours after initiation of Zn starvation in *S. luteus* mycelium, *SlZRT1* gene expression peaks and declines again to reach control levels after 4 h of growth in absence of Zn. After 24 h of growth in the absence of Zn, the *SlZRT1* expression level in *S. luteus* mycelium is slightly higher again compared to the level in mycelium grown in control conditions. These fluctuations in expression level could possibly reflect the cell’s Zn status. A similar expression pattern, though delayed in time was detected by Schothorst et al. (2017) in *S. cerevisiae* for *ScZRT1* in conditions of Zn deprivation. *ScZRT1* transcripts peak at 2 days under Zn deprivation and decline again afterward. A fast transcriptional response on limited environmental Zn concentrations is common for plasma membrane localized Zn transporters of the ZIP I subfamily. Induction of transcription in the absence of external Zn was reported previously for fungal ZIP I subfamily Zn importers which were identified in Ascomycota (ScZRT1 of *Schizosaccharomyces pombe*, ZrfA, ZrfB and ZrfC *of Aspergillus fumigatus*, Tzn1 and Tzn2 of *Neurospora crassa*, CaZRT1 and CaZRT2 of *Candida albicans*) and in Basidiomycota (CgZIP1 and GgZIP2 of *Cryptococcus gattii*) (Dainty et al., 2008; Kiranmayi et al., 2009; Jung, 2015).

Altogether, our data support a function as plasma membrane localized Zn importer with an important role in Zn homeostasis of *S. luteus* for SlZRT1. Likely, SlZRT1 is responsible for an adequate supply of Zn to the cell when environmental Zn is limited. With our current data, we cannot conclude on a role for SlZRT1 as Zn receptor for signaling in order to adjust primary metabolism to external Zn availability. Such a role was reported recently for ScZRT1 (Schothorst et al., 2017) and is certainly worth investigation in *S. luteus* and mycorrhizal fungi in general. Ectomycorrhizal fungi are well-known to offer their host plant a balanced nutrient supply by efficiently collecting limited nutrients and reducing the transfer of excess, potentially toxic elements. In relation to Zn, ectomycorrhizal fungi in general, and *S. luteus* in particular, are reported to protect their host plant from Zn toxicity (Colpaert et al., 2011). As trees in general do not tolerate high Zn soil concentrations, this protective feature of *S. luteus* is interesting for phytoremediation purposes. Further research is needed to better understand the regulation and function of *SlZRT1* within the *S. luteus* – host ectomycorrhizal association and to assess the contribution of SlZRT1 to the Zn status of the host plant.

## Author Contributions

LC, JR, and JC designed the study. LC, AT, ES, and NA performed the experiments. LC, EM, AK, and JR analyzed the data. LC and JR wrote the manuscript. LC, ES, NA, MODB, JR, and JC contributed in manuscript editing. All authors read and approved the final version of the manuscript.

## Conflict of Interest Statement

The authors declare that the research was conducted in the absence of any commercial or financial relationships that could be construed as a potential conflict of interest.

## Footnotes

1 http://genome.jgi.doe.gov/Suilu2/Suilu2.home.html

2 http://www.tcdb.org/

3 http://www.uniprot.org/

4 http://www.ncbi.nlm.nih.gov/

## Supplementary Material

FIGURE S1 Functional complementation of the zinc-uptake-deficient yeast strain Δzrt1Δzrt2 by SlZRT1::EGFP. Wild type (WT) and mutant yeast cultures with an OD_600_ = 1 were 10-fold serial diluted (10^0^, 10^-1^, 10^-2^, and 10^-3^) and spotted on control (1 mM Zn) or ethylenediaminetetraacetic acid (EDTA) supplemented synthetic drop-out (SD) medium. The WT strain was transformed with the empty vector (EV, pAG306GAL-ccdB-EGFP; Alberti et al., 2007), the mutant strain Δzrt1Δzrt2 with either the EV or the vector containing *SlZRT1:GFP*. The experiment was carried out for three independent clones and pictures were taken after 4 days of growth.

FIGURE S2 Fe concentration in transformed yeast cells **(A,B)**. The WT strain was transformed with the EV (pYES-DEST52, Invitrogen), the mutant strain with either the EV or the vector containing *SlZRT1*. Data are the average ± SE of five biological replicates, significant differences (*p* < 0.05) are indicated by different letters. **(A)** In control conditions (0 μm Zn), **(B)** after exposure to Zn (500 μM).
