# Supplementary figures and images for: The SlZRT1 Gene Encodes a Plasma Membrane-Located ZIP (Zrt-, Irt-Like Protein) Transporter in the Ectomycorrhizal Fungus Suillus luteus
Source: Front Microbiol. 2017 Nov 28;8:2320. doi: 10.3389/fmicb.2017.02320 (PMC5712335; doi:10.3389/fmicb.2017.02320)

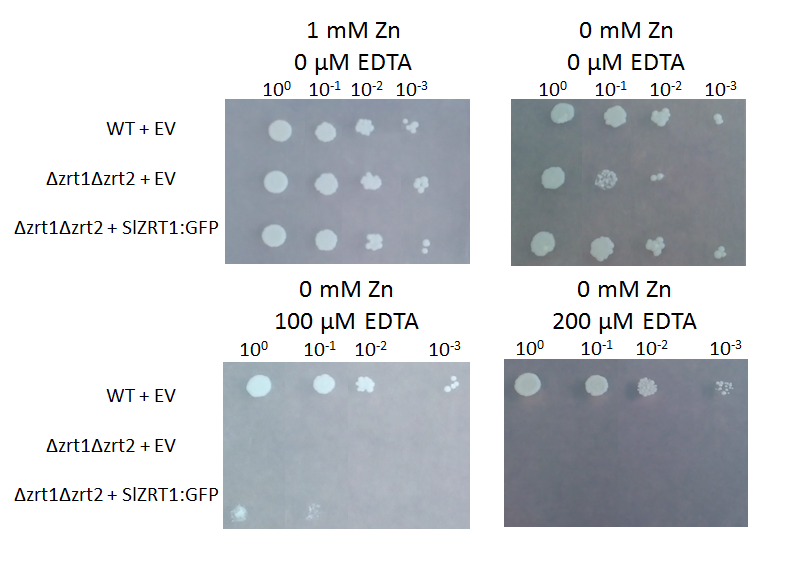

Supplement: FIGURE S1 — Functional complementation of the zinc-uptake-deficient yeast strain Δzrt1Δzrt2 by SlZRT1::EGFP. Wild type (WT) and mutant yeast cultures with an OD600 = 1 were 10-fold serial diluted (100, 10-1, 10-2, and 10-3) and spotted on control (1 mM Zn) or ethylenediaminetetraacetic acid (EDTA) supplemented synthetic drop-out (SD) medium. The WT strain was transformed with the empty vector (EV, pAG306GAL-ccdB-EGFP; Alberti et al., 2007), the mutant strain Δzrt1Δzrt2 with either the EV or the vector containing SlZRT1:GFP. The experiment was carried out for three independent clones and pictures were taken after 4 days of growth. [file Image_1.TIFF]

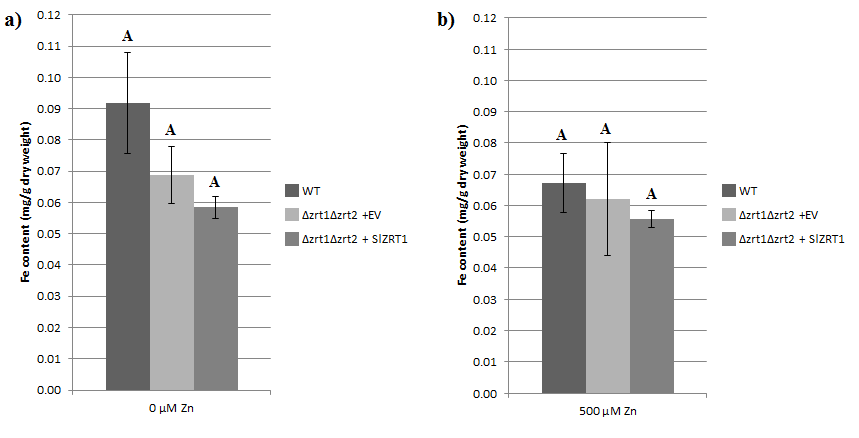

Supplement: FIGURE S2 — Fe concentration in transformed yeast cells (A,B). The WT strain was transformed with the EV (pYES-DEST52, Invitrogen), the mutant strain with either the EV or the vector containing SlZRT1. Data are the average ± SE of five biological replicates, significant differences (p < 0.05) are indicated by different letters. (A) In control conditions (0 μm Zn), (B) after exposure to Zn (500 μM). [file Image_2.TIF]
