# Supplementary material for: The SlZRT1 Gene Encodes a Plasma Membrane-Located ZIP (Zrt-, Irt-Like Protein) Transporter in the Ectomycorrhizal Fungus Suillus luteus
Source: Front Microbiol. 2017 Nov 28;8:2320. doi: 10.3389/fmicb.2017.02320 (PMC5712335; doi:10.3389/fmicb.2017.02320)
Supplement: Supplementary file 3 [file Table_1.DOCX]

**Supplementary Table 1.** **Characterized Fungal ZIP transporters**

| **Accession**  **number** | **Database** | **Protein** | **Gene** | **Organism** | **Status** |
| --- | --- | --- | --- | --- | --- |
| P32804 | Uniprot | Zinc-regulated transporter 1 | ZRT1 | *Saccharomyces cerevisiae*  (strain ATCC 204508 / S288c)  (Baker's yeast) | Reviewed |
| O94639 | Uniprot | Zinc-regulated transporter 1 | zrt1 | *Schizosaccharomyces pombe*  (strain 972 / ATCC 24843)  (Fission yeast) | Reviewed |
| Q12067 | Uniprot | Metal homeostasis factor ATX2 | ATX2 | *Saccharomyces cerevisiae*  (strain ATCC 204508 / S288c)  (Baker's yeast) | Reviewed |
| P40544 | Uniprot | Zinc transporter YKE4 | YKE4 | *Saccharomyces cerevisiae*  (strain ATCC 204508 / S288c)  (Baker's yeast) | Reviewed |
| P34240 | Uniprot | Zinc-regulated transporter 3 | ZRT3 | *Saccharomyces cerevisiae*  (strain ATCC 204508 / S288c)  (Baker's yeast) | Reviewed |
| O94402 | Uniprot | Probable zinc transporter zip2 | zip2 | *Schizosaccharomyces pombe*  (strain 972 / ATCC 24843)  (Fission yeast) | Reviewed |
| Q12436 | Uniprot | Zinc-regulated transporter 2 | ZRT2 | *Saccharomyces cerevisiae*  (strain ATCC 204508 / S288c)  (Baker's yeast) | Reviewed |
| 180140 | JGI | ZIP-like iron-zinc transporter | ZIP-A | *Laccaria bicolor* v2.0 | Described by  Doillon 2010^a^ |
| 305445 | JGI | ZIP-like iron-zinc transporter | ZIP-B | *Laccaria bicolor* v2.0 | Described by  Doillon 2010^a^ |
| 309863 | JGI | ZIP-like iron-zinc transporter | ZIP-C | *Laccaria bicolor* v2.0 | Described by  Doillon 2010^a^ |
| 189929 | JGI | ZIP-like iron-zinc transporter | ZIP-D | *Laccaria bicolor* v2.0 | Described by  Doillon 2010^a^ |
| 309134 | JGI | ZIP-like iron-zinc transporter | ZIP-E | *Laccaria bicolor* v2.0 | Described by  Doillon 2010^a^ |
| ^a^ Doillon, D. (2010). Déterminants moléculaires de la tolérance au zinc des microorganismes eucaryotes (Thèse de doctorat, Université Henri Poincaré (Nancy 1), FRA). | | | | | |
